# What are the barriers to access to mental healthcare and the primary needs of asylum seekers? A survey of mental health caregivers and primary care workers

**DOI:** 10.1186/s12888-016-1048-6

**Published:** 2016-09-29

**Authors:** Javier Bartolomei, Rachel Baeriswyl-Cottin, David Framorando, Filip Kasina, Natacha Premand, Ariel Eytan, Yasser Khazaal

**Affiliations:** 1 CAPPI Servette, Department of Mental Health and Psychiatry, Geneva University Hospitals, 91 rue de Lyon, CH-1203 Genève, Switzerland; 2 Department of Mental Health and Psychiatry, Geneva University Hospitals, 2 Ch du Petit Bel-Air, CH-1225 Chêne-Bourg, Switzerland; 3 Department of Mental Health and Psychiatry, Substance Abuse Unit, Geneva University Hospitals, Genève, Switzerland

**Keywords:** Asylum seekers, Priority needs, Barriers to access to mental health services

## Abstract

**Background:**

We aimed to assess the opinion of primary care workers, social workers, translators and mental health caregivers who work with asylum seekers about the latter’s unmet needs and barriers to access to mental healthcare.

**Methods:**

We used a Likert scale to assess the opinion of 135 primary care workers (general practitioners, nurses, social workers and translators) and mental health caregivers about the proportion of asylum seekers with psychiatric disorders, their priority needs and their main barriers to mental health services.

**Results:**

Insufficient access to adequate financial resources, poor housing and security conditions, access to employment, professional training and legal aid were considered as priority needs, as were access to dental and mental healthcare. The main barriers to access to mental healthcare for asylum seekers included a negative representation of psychiatry, fear of being stigmatized by their own community and poor information about existing psychiatric services.

**Conclusions:**

We found a good correlation between the needs reported by healthcare providers and those expressed by the asylum-seeking population in different studies. We discuss the need for greater mobility and accessibility to psychiatric services among this population.

## Background

Asylum seekers represent a particularly vulnerable segment of the population, due in part to the numerous stressful events to which they are exposed [1–4]. These events may explain the increased rates of anxiety disorders, post-traumatic stress disorder and depression among this group [5–7].

Despite these probable important mental health needs, most asylum seekers do not consult mental health services [8–11]. Numerous unmet needs within this population have been reported, such as housing, food, school fees, and medical and dental care [8]. In Switzerland, 45,602 people (i.e., Eritrean 16.4 %, Syrian 13.2 %, Afghanistani 8.3 %) were applying for asylum in July 2014, 6 % of these in the Canton of Geneva [12]. People who are authorized to work include those who have a provisional refugee license (F permit) or who hold an N permit: Asylum seekers with an N permit are persons who have applied for asylum in Switzerland and whose application is being processed; during the asylum proceeding, they are basically entitled to be resident in Switzerland and are allowed under certain circumstances and after at least 3 months to pursue gainful employment as an employee subject to Article 43 of the Asylum Act. Only 10.3 % of those with an N permit and 24.1 % of those with a provisional license were actively employed. Collecting more detailed data in this field at the Swiss level is necessary [13], and understanding the views of caregivers regarding the needs of asylum seekers may help improve the services offered to such a population. Previous studies [14] have pointed out that services rarely offer opportunities to refugees to express their basic needs, or how the institutional responses to these needs are different from what is expected by this population, increasing the risk of mistrust [15, 16].

In the present study carried out in Geneva, we aimed to assess the opinions of primary care workers (general practitioners [GPs], nurses, social workers, translators) and mental health caregivers who work with asylum seekers about the latter’s unmet needs and the possible barriers to access to mental healthcare. The type of medical speciality (primary caregiver versus mental health caregiver), years of experience with this population (more or less than 5 years) and frequency of contact with asylum seekers were assessed for their impact on the participants’ answers.

## Methods

### Setting

All questioned participants were in contact with asylum seekers in different contexts. When arriving in Switzerland, each asylum-seeking person is assigned a GP in “a gatekeeping system”. Moreover, each asylum seeker is lodged in a group home, where global health nurses provide systematic health evaluations. When psychiatric evaluation is needed, the GP can refer the patient to the Transcultural Psychiatry Program of the Geneva University Hospitals, which decides what kind of psychiatric care is appropriate [17]. Clinical consultations are translated by an interpreter of the Red Cross Interpretation Service.

### Population studied

Included participants were recruited among GPs (32) working in a public outpatient transcultural program or in private practice; psychiatrists (21) and psychologists (4) working in public outpatient and inpatient units, or in non-governmental organizations specialized in the psychosocial treatment of migrants (psychiatrists and psychologists in private practice in Geneva rarely treat asylum seekers); nurses specialized in somatic care (10) working in group homes; and mental health nurses (47) working in public outpatient and inpatient units. We also included social workers (7) involved in socio-legal assistance, educators (1) and translators (7) from the Red Cross, and miscellaneous professions involved in psychiatric care of asylum seekers (occupational therapists, psychomotor therapists, *N* = 7).

### Recruitment

All participants received three professional emails inviting them to participate in this study that included information about its goals and procedures. The responder rate was 64 % for primary care physicians (32 responders, 18 non-responders), 83.3 % for general care nurses (10 responders, 2 non-responders), 15 % for translators (6 responders, 34 non-responders) and less than 3 % for social workers (4 responders in the psychiatric field and 3 responders in the non-psychiatric field). Among mental health caregivers, the response rate was 42 % for psychiatrists (21 responders and 29 non-responders), 27.3 % for specialized mental health nurses (47 responders and 123 non-responders) and less than 5 % for psychologists (4 responders). Moreover, there was no possibility for non-responders to explain why they declined.

### Data collection

A questionnaire was created specifically for this study. Participants answered it anonymously after giving their online informed consent by using a “Survey Monkey” link. Each participant could express his or her agreement or disagreement with each proposed assertion by using five Likert scale options: “disagree”,” mostly disagree”, “mostly agree”, “somewhat agree” and “agree”.

The questionnaire was divided into the following parts (Table 1):

**Table 1:** Questionnaire

1. Socio-demographic and professional characteristics:	
Age range	Number of years spent working with AS
Gender	Amount of time spent working directly with AS during an average workday
Occupation	
2. Subjective assessment of proportion of AS with mental health problems:	
*What is the prevalence of psychiatric illness amongst the AS population*?	*What proportions of AS are receiving appropriate mental health care*?
3. Priority needs of AS: *In your opinion*, *do asylum seekers have*…	
*adequate financial resources*?	*access to means of communication to be able to contact relatives living in their countries of origin* ?
*adequate means to assure appropriate hygiene*?	
*adequate means to assure appropriate nutrition*?	*access to secure living conditions*?
*good access to schooling for their children*?	*access to public transportation*?
*access to appropriate housing conditions*?	*access to the practice of religion*?
*In your opinion*, *a priority need for asylum seekers is*…	
*to increase access to employment*?	*to improve access to somatic care*?
*to increase access to vocational training*?	*to improve access to dental care*?
*to increase access to legal aid*?	*to improve access to psychiatric care*?
*to improve housing conditions*?	
4. Barriers to access to mental healthcare: *In your opinion*, *what are the reasons for not consulting*?	
*Trivializing of mental suffering*	*Fear of lack of confidentiality of consultations*
*Negative opinion of psychiatry*	*Previous negative psychiatric experience*
*Fear of being stigmatized by one*’*s community*	*Fear of receiving medication with side effects*
*Lack of information on the existing mental health care services available*	*Fear of involuntary hospitalization*
	*Fear of being penalized in one*’*s application for asylum*
*Complexity of the healthcare network*	*Fear of being discriminated against by caregivers*

*Abbreviation: AS* asylum seekers

1. Socio-demographic and professional characteristics of the participants.
2. Subjective assessment of the proportion of asylum seekers who had mental health problems without receiving proper care.
3. Views on the needs of asylum seekers.
4. Views on the barriers to access to mental healthcare for this population. At the end of the questionnaire, respondents had the opportunity to add a personal comment.

Answer options were grouped as follows: “disagree and mostly disagree” percentages on one side (corresponding to “disagree”), and “mostly agree” and “agree” on the other.

### Statistics

Descriptive statistics were used for the three parts of the questionnaire. In addition, we compared the responses of the subjects in terms of their characteristics by using a chi-square test. All distributions were controlled and normally distributed. Statistics were computed with SPSS software, version 22 (IBM Corporation, Armonk, NY, USA).

For answers corresponding to the highest “disagree” or “agree” percentages, we performed a chi-square test to determine whether there were significant differences in perception between:

- primary caregivers and mental health caregivers
- caregivers working for more than 5 years with asylum seekers and those working for less than 5 years
- caregivers who worked rarely or occasionally with this population and those who worked frequently or exclusively with it

## Results

### Participation rate and socio-demographic data (Table 2)

From 1 March 2014 to 30 August 2014, we collected answers from 135 professionals who all had contact with asylum-seeking patients. Respondents were mostly women (62.3 %) and mental healthcare providers (60 %), with a strong representation of physicians (39.3 %) and nurses (42.2 %). Of the total group, 64.2 % were over 40 years old, 56.7 % worked for at least 5 years with asylum seekers, 54.1 % reported working frequently with this population and 34.6 % reported working occasionally with them. In addition, 66.6 % estimated that the prevalence of psychiatric illness in this population was between 20 % and 60 % (67.9 % of physicians and 42.1 % of nurses), and 75.9 % estimated that only 20 to 40 % of asylum seekers received appropriate mental healthcare.

**Table 2:** Characteristics of caregivers

Workplace	
Psychiatric care	60 %
Non-psychiatric care	40 %
Profession (*N* = 135)	
Physician	39.3 %
Psychologist	3 %
Nurse	42.2 %
Social worker	5.2 %
Educator	0.7 %
Translator	4.4 %
Other	5.2 %
Gender (*N* = 130)	
Male	37.7 %
Female	62.3 %
Age (*N* = 134)	
Less than 30	8.2 %
Between 30 and 40	27.6 %
Between 40 and 50	26.9 %
Older than 50	37.3 %
Number of years spent working with asylum seekers (*N* = 134)	
Less than 2 years	18.7 %
Between 2 and 5 years	24.6 %
Between 5 and 10 years	19.4 %
More than 10 years	37.3 %
Number of years spent working with asylum seekers (*N* = 134)	
Less than 5 years	43.3 %
More than 5 years	56.7 %
Time spent working directly with asylum seekers during an average workday (*N* = 133)	
Rare	11.3 %
Occasionally	34.6 %
Frequently	45.9 %
Exclusively	8.3 %
Time spent working directly with asylum seekers during an average workday (*N* = 133)	
Rare and Occasionally	45.9 %
Frequently and Exclusively	54.1 %
What is the prevalence of psychiatric illness amongst the asylum-seeking population? (*N* = 132)	
Approximately 2 of 10	20.5 %
Between 2 and 4 of 10	37.1 %
Between 4 and 6 of 10	29.5 %
Between 6 and 8 of 10	12.9 %
What proportions of asylum seekers are receiving appropriate mental healthcare? (*N* = 133)	
Approximately 2 of 10	39.8 %
Between 2 and 4 of 10	36.1 %
Between 4 and 6 of 10	18.8 %
Between 6 and 8 of 10	5.3 %

### Priority needs (Tables 3 and 4)

We found that 71.9 % of respondents estimated that asylum seekers had insufficient access to adequate financial resources, 79.3 % that they had insufficient access to housing conditions and 71.4 % that they had insufficient access to adequate security. Access to employment (85.8 %), vocational training (70.4 %) and legal aid (85.7 %), as well as improvement of housing conditions (85.9 %) were considered as priority needs. Access to dental care (71.1 %) and mental healthcare were also considered as priority needs (75.4 %). On the other hand, answers regarding access to proper hygiene, food, schooling for children and public transport; freedom to practice one’s religion; and access to somatic care had a more heterogeneous distribution without as clear a tendency.

**Table 3:** Priority needs of asylum seekers

Questions: In your opinion, do asylum seekers have	Disagree	Mostly agree	Agree
Adequate financial resources? (*N* = 135)	71.9 %	20 %	8.1 %
Adequate means to ensure appropriate hygiene? (*N* = 133)	33.1 %	47.4 %	19.5 %
Adequate means to ensure appropriate nutrition? (*N* = 133)	60.9 %	29.3 %	9.8 %
Good access to schooling for their children? (*N* = 134)	21.6 %	31.3 %	47 %
Access to appropriate housing conditions? (*N* = 135)	79.3 %	20 %	0.7 %
Access to a means of communication to contact relatives living in their countries of origin? (*N* = 135)	62.2 %	35.6 %	2.2 %
Access to secure living conditions? (*N* = 133)	71.4 %	20.3 %	8.3 %
Access to public transportation? (*N* = 133)	27.8 %	23.3 %	48.9 %
Access to the practice of religion? (*N* = 131)	11.5 %	29.8 %	58.8 %

**Table 4:** Priority needs of asylum seekers

Questions: In your opinion, a priority need for asylum seekers is	Disagree	Mostly agree	Agree
To increase access to employment? (*N* = 134)	3.7 %	10.4 %	85.8 %
To increase access to vocational training? (*N* = 135)	8.9 %	20.7 %	70.4 %
To increase access to legal aid? (*N* = 133)	2.3 %	12 %	85.7 %
To improve housing conditions? (*N* = 135)	1.5 %	12.6 %	85.9 %
To improve access to somatic care? (*N* = 133)	24.1 %	18.8 %	57.1 %
To improve access to dental care? (*N* = 135)	8.9 %	20 %	71.1 %
To improve access to psychiatric care? (*N* = 134)	9 %	15.7 %	75.4 %

### Barriers to access to mental healthcare (Table 5)

Among the barriers to access to mental healthcare considered to be important by the respondents were negative representations of psychiatry (65.7 %), fear of being stigmatized by asylum seekers’ own community (66.4 %) and lack of information about the existing mental healthcare services available (66.2 %). Trivializing of mental suffering, complexity of access to healthcare services, fear of lack of confidentiality, prior experiences in mental healthcare, fear of medication and side effects, fear of involuntary hospitalization, penalization of the asylum application for receiving mental healthcare and fear of being stigmatized by caregivers were not as clearly considered to be barriers to access to mental healthcare.

**Table 5:** Barriers to access to mental healthcare

Reasons for not consulting	Not at all	A little	A lot
Trivializing of mental suffering (*N* = 135)	39.3 %	31.1 %	29.6 %
Negative opinion of psychiatry (*N* = 134)	11.2 %	23.1 %	65.7 %
Fear of being stigmatized by one’s community (*N* = 134)	6.7 %	26.9 %	66.4 %
Lack of information on the existing mental healthcare services available (*N* = 133)	14.3 %	19.5 %	66.2 %
Complexity of the healthcare network (*N* = 135)	22.2 %	31.9 %	45.9 %
Fear of lack of confidentiality of consultations (*N* = 135)	24.4 %	28.9 %	46.7 %
Previous negative psychiatric experiences (*N* = 133)	49.6 %	31.6 %	18.8 %
Fear of receiving medication with side effects (*N* = 133)	44.4 %	30.8 %	24.8 %
Fear of involuntary hospitalization (*N* = 133)	51.9 %	24.1 %	24.1 %
Fear of being penalized in one’s application for asylum (*N* = 133)	51.9 %	21.8 %	26.3 %
Fear of being discriminated against by caregivers (*N* = 135)	65.2 %	22.2 %	12.6 %

### Perception of needs and barriers to treatment in terms of socio-demographic data

#### Perception of percentage of asylum seekers receiving appropriate care

Among the respondents, 85 % of mental health professionals versus 61.3 % of primary caregivers estimated that only 20 to 40 % of asylum seekers with mental health problems were receiving appropriate care (*p* < 0.001). We also found significant differences in the perception of appropriate care in terms of the frequency of contact with asylum seekers: 22.9 % of those working frequently with them versus 59 % of those working rarely or occasionally with them estimated that 20 % of asylum seekers were receiving appropriate care (*p* < 0.001). We did not find any significant differences concerning appropriate mental healthcare between professionals having worked more or less than 5 years with this population.

### Primary needs

On the issues of access to adequate financial resources, housing conditions and low-paid employment, we did not find significant differences based on the type, duration or frequency of work with this population. Similarly, we did not find significant differences in the perception of the need to improve access to legal aid or to dental care. Regarding access to safe housing conditions, however, we found a significant difference of opinion depending on the different caregivers’ work; the group of caregivers in mental health (83.3 %) believed there to be significantly worse safety conditions than did the group in primary care (52.8 %) (*p* < 0.001). On the issue of access to unpaid training, we found a significant difference in perception depending on the frequency of work (*p* < 0.01) with the asylum-seeking population; those who frequently or exclusively worked with this population (83.3 %) were more likely to define this point as a priority than were those who rarely worked with this population (55.7 %). Finally, on the subject of access to mental healthcare, with a marginally significant difference (*p* = 0.05), mental health caregivers (82.5 %) considered this to be a priority need more often than primary caregivers did (64.8 %).

### Care barriers

We observed that 65.3 % of the respondents agreed strongly or very strongly that the negative portrayal of psychiatry is a major barrier to access to mental healthcare. We did not find any significant differences in this perception related to the three variables we studied: type, duration or frequency of work. Similarly, 66 % agreed strongly or very strongly that the fear of being stigmatized by one’s community is a significant barrier to access to mental healthcare, again without any significant differences related to the three variables we studied. Mental healthcare providers (72.2 %), however, believed more strongly than their primary care counterparts (57.4 %) that the lack of information about mental healthcare services available represents a significant barrier to patients seeking treatment (*p* < 0.05). Caregivers who work rarely or occasionally (77 %) with this population also believed, more strongly than did those who work with them frequently or exclusively (57.1 %), that this lack of information represents a barrier (*p* = .01).

## Discussion

In this study, we aimed to assess primary care workers’ and mental health caregivers’ opinions about the needs of asylum seekers and the factors that may explain their underutilization of psychiatric services. To our knowledge, this is the first study of its kind in Switzerland.

### Estimation of the prevalence of mental disorders

Our results showed that 66.6 % of the respondents believed that the prevalence of psychiatric disorders in asylum seekers is between 20 % and 60 %, but 74.9 % of the respondents believed that only 20 to 40 % of asylum seekers receive adequate mental healthcare. Mental health caregivers believed that a lower percentage of asylum seekers receive this care (85 % of them think that only 20 to 40 % of asylum seekers receive adequate psychiatric care) than primary care caregivers did (69.8 % of them think that 20 to 60 % of asylum seekers receive appropriate psychiatric care). A greater sensitivity to these issues is possibly due to their regular involvement with patients who received adequate psychiatric care too late, often in acute settings such as a hospital or a crisis centre. These findings are consistent with a known risk of under-diagnosis of mental health disorders within this population [18–20].

### Perception of priority needs and barriers to access to psychiatric treatment

The main reported needs are related to adequate housing conditions and security, as well as access to sufficient financial resources, paid work, legal assistance, and psychiatric and dental care. These findings are similar to those reported in studies of asylum seekers and refugees [8, 19, 21]. The need for security was not, however, reported in the previous studies. This is probably due to the perception of caregivers, especially mental health caregivers, of the violent conditions that asylum seekers live in. Access to professional training was perceived to be a more significant priority by the population of caregivers working exclusively or frequently with this population, which could be explained by their greater awareness of this issue through close contact.

The main barriers to access to mental healthcare services identified in the study are a negative portrayal of psychiatry, fear of being stigmatized by one’s community and lack of information concerning the psychiatric services available. GPs working with asylum seekers and refugees mentioned their low utilization of medical services [22]. Moreover, a low level of education, along with difficulties with the local language, appeared to be the main barriers to access to somatic care.

Different studies have pointed out that asylum seekers are generally reluctant to seek mental health care despite a high level of psychological distress [20], that they consult mental health services far less than do the local populations [23] and that they try to shorten the duration of their hospital stays as much as possible [24]. In our study, a negative opinion of psychiatry and a fear of being stigmatized by one’s community were considered to be two of the three main barriers to access to mental care services. These results are consistent with studies that have highlighted how representation of psychiatric symptoms can differ from one culture to another and how traditional solutions may be preferred to a psychiatric approach (such as seeking help from a traditional healer, religious leader or elder member of the ethnic community) [9, 10, 25].

Lack of information regarding existing healthcare services was identified as a significant barrier to care by most respondents, which is consistent with the findings from previous studies [23, 26]. The distance between the asylum seeker’s residence and the care facility is also a potential barrier to care, especially when individuals have no access to public transportation or believe that they do not have access [26]. In our study, access to public transport was not considered as a priority need (Table 2), even though many of the group homes are located on the outskirts of the city. General care nurses who work in group homes therefore have a crucial role in detecting mental disorders and in providing information about access to psychiatric services [17]. In our study, mental health caregivers (72.2 % of them agree with this proposition versus 57.7 % of primary caregivers, with a global distribution difference of *p* = 0.02) felt more strongly that lack of information was a barrier to access to care, possibly because of their own knowledge of the complexity of the mental healthcare service network and the consequences they observe when there are delays in consulting a healthcare professional for this population. People working only rarely or occasionally with the asylum-seeking population also felt strongly that lack of information could be a strong barrier to access to treatment.

Lack of access to a translator is also mentioned in the literature as a major barrier to care [27]. In consideration of the systematic use of translators in Swiss mental healthcare, we did not specifically investigate this item, but respondents were able to describe such a barrier via the open-ended comments. We did not find any significant mention of the lack of access to translators as being a barrier to access to care.

### Limitations

Little research has been performed about the experiences and perception of mental health professionals in psychiatric services in Europe [15]. However, not asking the asylum seekers themselves about their essential needs and their barriers to access to psychiatric services is a limitation of our study. Asylum seekers seldom express themselves about their needs [8] and there is a real therapeutic interest in offering them a specific means of expression [28]. Further studies should compare refugees’ and caregivers’ perceptions of primary needs and barriers to access to treatment in order to improve the efficiency of and access to our services. The moderate number of responses, along with the heterogeneity of participation rates among different professional groups, limits the generalizability of the present results. Finally, our study could not specify the impact of cultural influence on primary needs and barriers to access to services, which may be of interest [29].

## Conclusion

To our knowledge, this is the first study in Switzerland to collect and compare the assessment of priority needs of asylum seekers and the barriers to access to mental healthcare from the point of view of global and mental healthcare providers. Our study highlights that there exists a good correlation between the needs reported by healthcare providers and those expressed by asylum seekers themselves in different studies. The results underline the perceived links between poor housing conditions and a lack of professional or educational activity for mental health [30].

Different European countries are currently seeking a consensus on the principles of good practice in the domain of healthcare for immigrants, principles that imply easy and equal access [31]. In the present study, lack of information on existing structures is considered to be one of the main barriers. Further studies should assess the impact of different measures on an increase in access to mental healthcare by asylum seekers (i.e., psychiatric availability at the residences of asylum seekers, closer collaboration with voluntary organizations and local key people such as religious and community leaders, etc.).

## Abbreviation

GP: General practitioner
